# 3D-Printed Versus Conventional Dental Provisional Resins: A Comparative Study

**DOI:** 10.3390/medicina62020382

**Published:** 2026-02-14

**Authors:** Olívia Breda Moss, Anselmo Agostinho Simionato, Adriana Cláudia Lapria Faria, Renata Cristina Silveira Rodrigues, Ricardo Faria Ribeiro

**Affiliations:** Department of Dental Materials and Prostheses, School of Dentistry of Ribeirão Preto, University of São Paulo, Café Avenue SN, Ribeirão Preto 14040-904, SP, Brazil; oliviabm@usp.br (O.B.M.); anselmo.simionato@usp.br (A.A.S.); adriclalf@forp.usp.br (A.C.L.F.)

**Keywords:** 3D printing, computer-aided manufacturing, dental materials, acrylic resins

## Abstract

*Background and Objectives:* This study aimed to evaluate and compare the effects of immersion and brushing on resins used for temporary crowns, including two 3D-printed resins (Nanolab and PrintaX) and one self-curing resin (Duralay), with different surface finishing protocols. *Materials and Methods:* Printed specimens were designed using specialized software, followed by slicing and printing. Self-curing resin samples were fabricated using silicone matrices, with the printed specimens serving as references. Square samples (7.0 × 7.0 × 2.0 mm, *n* = 90) were divided into three groups based on surface finishing: extrinsic pigment with glaze, glaze only, and polish only. The samples were immersed in 15 mL of cola soft drink, energy drink, or distilled water for six days at 37 °C in a dark environment before undergoing a brushing test (180 cycles/minute, 65,700 cycles, 2 N, 37 °C). Color alterations, surface roughness, and Knoop microhardness were then analyzed. *Results:* Statistical analyses revealed that all factors significantly influenced the tested properties (*p* < 0.05). Nanolab exhibited the most pronounced color alterations, with ∆E_00_ values reaching up to 22.21 ± 3.13 in specific conditions (e.g., Glaze, Cola soft drink). It also presented increased surface roughness, particularly when compared to PrintaX. Conversely, Duralay consistently displayed the highest Knoop microhardness changes (e.g., ranging from −1.84 ± 0.36 to 0.47 ± 0.45 in different conditions) across most experimental groups. Polishing consistently provided better outcomes in terms of color stability, surface roughness, and microhardness compared to extrinsic pigment + glaze or glaze-only treatments. The first immersion generally led to the greatest color change. *Conclusions:* The acidic challenge promoted significant changes in the optical and surface properties of the evaluated resins, increasing ∆E_00_ and roughness and reducing microhardness to different extents depending on the material. Clinically, these findings highlight the relevance of material selection and limiting exposure to acidic beverages during provisional use.

## 1. Introduction

Technological advancements have driven the widespread adoption of computer-aided design (CAD) and computer-aided manufacturing (CAM) systems, leading to the development of new materials and equipment for digitization and workflow automation [1,2]. These manufacturing processes can be broadly classified into subtractive manufacturing, which involves milling, and additive manufacturing, which encompasses 3D printing [3].

Additive manufacturing enables the transformation of virtual three-dimensional 40 models into precise and efficient physical prototypes. Advances in processing methods and material science have contributed to increasing applications across various industries, including dentistry [4,5]. In the dental field, 3D printing facilitates the production of patient-specific models, allowing for the fabrication of ready-to-use components with reduced material waste compared to subtractive techniques [4,6]. This technology is utilized in the manufacturing of surgical guides, dental models, provisional crowns, artificial teeth, and prosthetic bases [7,8].

Provisional restorations play a critical role in rehabilitative dental treatments by restoring aesthetics, function, and protection to prepared teeth while ensuring predictability for the final restoration [9,10]. The indirect fabrication of these restorations—digitally designed and produced layer by layer—is regarded as a high-quality, safe, and efficient alternative to direct procedures, offering advantages such as increased precision, cost-effectiveness, and reduced material waste [11]. When selecting a material for provisional restorations, key parameters such as color stability, microhardness, and surface roughness must be considered, even for short-term applications [12,13].

Polymethyl methacrylate (PMMA) has been widely regarded as the standard material for provisional prostheses and crowns over the past decades [14]. However, it requires extensive laboratory and clinical time for adjustments and polishing [15,16,17,18] and presents limitations, including polymerization shrinkage, surface porosity, and susceptibility to discoloration [19].

The optimal polishing method for these materials remains a subject of investigation, with studies evaluating the effects of polishing, glazing, or no surface treatment [5,20]. However, the literature lacks consensus regarding the behavior of 3D-printed temporary resins compared to milled and conventional resins.

This study aimed to evaluate color stability, microhardness, and surface roughness in both 3D-printed and conventional temporary resins following different surface treatments, immersion in various solutions, and brushing. The null hypothesis tested was that finishing type, immersion, and brushing would not significantly affect the properties of the tested resins.

## 2. Materials and Methods

### 2.1. Materials Tested

**Figure 1:**
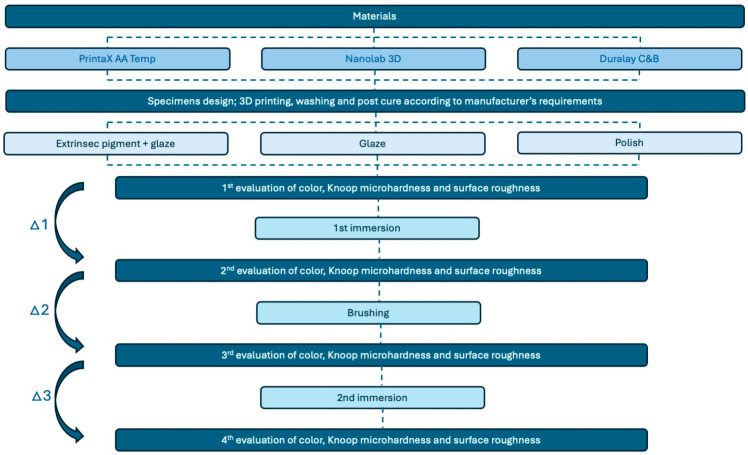
Flowchart of the experimental design, selected materials and evaluated properties.

The study was conducted from 2022 to 2024. For this study, three resins with indications for provisional restoration were used, two for fabrication on a digital light processing (DLP) 3D printer: PrintaX AA Temp (Odontomega, Ribeirão Preto, SP, Brazil) and Nanolab 3D (Wilcos do Brasil Ltd., Petrópolis, RJ, Brazil); and a gold standard self-curing resin as a control: Duralay (Reliance Dental Mfg., LLC, Worth, IL, USA) (Table 1). Before the impression process, the resin vials were shaken for 2 min to ensure homogenization of the material. A schematic flowchart of the sample processing and experimental process is shown in Figure 1.

### 2.2. Obtaining the Specimens

**Figure 2:**
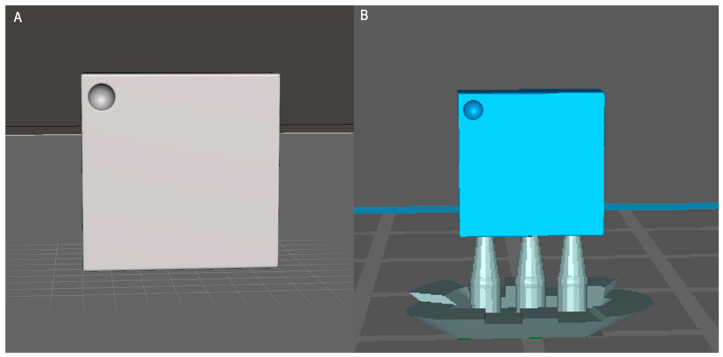
(**A**) Sample in the Meshmixer software; (**B**) Sample in Chitubox software.

The CAD/CAM specimens were designed using drawing software (Meshmixer v. 3.5.474, Autodesk Inc., San Francisco, CA, USA) (Figure 2A), generating a file in Standard Tessellation Language (.stl) format, square (7.0 mm × 7.0 mm × 2.0 mm). To ensure the standardization of the readings and the immersion and brushing tests, a circular marking located in the upper left quadrant of the surface opposite to where the finishings were applied was added during the sample-taking stage. These files were then exported to slicing software (CHITUBOX Basic 1.9.1, CBD Technology Co., Shenzhen, China) (Figure 2B), where the supports were added and the printing parameters indicated by the manufacturers were determined (Table 2).

After the impression, the excess resin was removed and the samples were bathed in isopropyl alcohol for 10 min under agitation (Tornado 3D Cleaning System, Odontomega, Ribeirão Preto, SP, Brazil) to remove uncured monomers remaining on the surface, with the alcohol being changed between baths, and dried at room temperature. The post-curing process was carried out by exposing the samples to UV light (Photo Phrozen Cure V2 Oven, Odontomega, Ribeirão Preto, Brazil), with the exposure time recommended by the manufacturers: PrintaX—3 cycles of 1 min, and Nanolab—1 cycle of 15 min.

For conventional resin, a printed sample was used to make a condensation silicone matrix (Zetalabor, Zhermack, Rovigo, Italy), standardizing the process of obtaining the specimens. A circular marking was also added to the surface opposite the surface where the finishes were applied, located in the upper left quadrant, to ensure standardization of the initial reading and after the immersion and brushing tests.

All the samples were polished with 600, 1200 and 2000 grit wet sandpaper, their dimensions were measured with a digital caliper (Mitutoyo Sul Americana Ltd., Suzano, Brazil) and they were cleaned in distilled water under ultrasonic agitation for 10 min to remove any remaining material and dried at room temperature.

### 2.3. Characterization of the Surface of the Test Specimens

**Figure 3:**
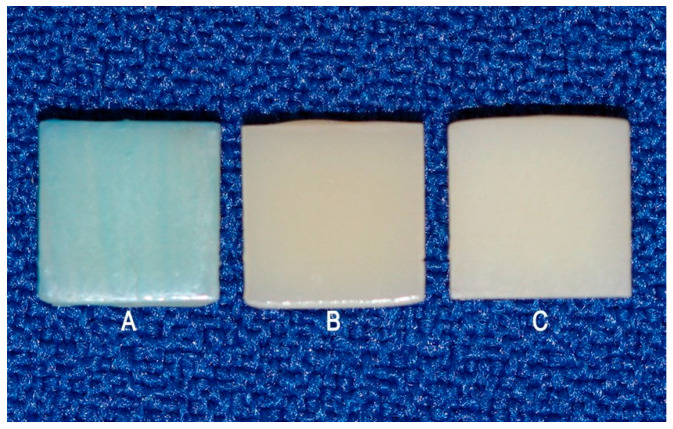
Appearance of the samples after surface finishing: (**A**) Extrinsic pigment + glaze; (**B**) Glaze; (**C**) Polishing.

The samples were randomly divided into three subgroups (n = 10) with different surface treatments: PG—extrinsic pigment + glaze; G—glaze; and POL—polishing. The ex- trinsic pigment + glaze (PG) subgroup received a layer of extrinsic Blue pigment (Cosmos Creation Kit, Yller, Pelotas, RS, Brazil), which was light-cured for 1 min in a UV light chamber, and a layer of glaze (PriZma SEAL, Makertech Labs, Tatuí, SP, Brazil) which acted for 10 s and was polymerized for 15 min in a UV light chamber. Characterization with pigment and glaze simulates the need to adjust the shade of the provisional restoration. The blue pigment was chosen to prevent the pigment in the immersion solutions from masking or exacerbating the color changes. The glaze subgroup (G) received only the glaze layer and, after 10 s, light-curing for 15 min. The polishing subgroup (POL) was only polished as early informed (Figure 3). In all applications, soft brushes were used in a single direction, avoiding bubbles, and following the manufacturers’ recommendations.

The sample size for this study was established based on previous studies in the literature on 3D printed provisional restorations, adopting 10 samples per subgroup [21,22]. The samples were evaluated for color, Knoop microhardness and surface roughness at four points in time: initial reading, after the first immersion, after brushing and after the second immersion.

### 2.4. Immersion in Solutions

The samples were immersed in 15 mL of each solution: cola soft drink (Coca-Cola Original, Rio de Janeiro Refrescos LTDA, Rio de Janeiro, RJ, Brazil), energy drink (Red Bull Energy Drink, Rauch Fruchtsafte GmbH & Co OG, Rankweil, Austria) and distilled water, which did not require any preparation prior to the test, for 06 days at 37 °C in a dark environment, with the solutions changed daily and stirred every 12 h to avoid decantation of the components. The total immersion time was 144 h, simulating 6 months of consumption [23]. At the end of the test, the samples were removed from the solutions, washed in distilled water and dried for subsequent analysis. This test was carried out in two stages according to the same criteria. The cola soft drink and the energy drink were selected as staining solutions to simulate frequent dietary exposure to highly acidic (Cola soft drink pH ≈ 2.37; Energy drink pH ≈ 3.30) and pigmented beverages, providing a clinically relevant assessment of the resins’ long-term color stability.

### 2.5. Brushing Simulation

For brushing, soft toothbrushes (Johnson’s Eco, Johnson & Johnson Ltd., São José dos Campos, Brazil) were used, positioned in the XY brushing machine (Biopdi, São Carlos, Brazil), set at a speed of 180 cycles/minute, for 65,700 cycles, a load of 2 N and a temperature of 37 °C. The solutions were prepared in a ratio of 2:1, 150 g of dentifrice (Colgate Total 12 Clean Mint, Colgate-Palmolive Ind. e Com. Ltd., São Paulo, Brazil) and 300 mL of distilled water, using a vacuum spatulator for 02 min. This test simulated 06 months of brushing, considering that a patient performs 02 daily brushing sessions of 02 min [24,25]. After completion, the samples were cleaned in distilled water, dried at room temperature and stored for subsequent analysis.

### 2.6. Evaluation of Color Change

A Delta Vista 450G spectrophotometer (Delta Color Ind. e Com. Equip. Ltd.a, São Leopoldo, RS, Brazil) was used to determine color change. For each sample, at each stage of analysis, a single measurement was taken in the central region of the sample by the same calibrated operator. To simulate natural daylight, the samples were evaluated under standard D65 lighting conditions. The L*, a* and b* coordinate values were recorded and entered the CIEDE2000 color difference formula [26,27]:

$${∆E}_{00}=\sqrt{{\left(\frac{{∆L}^{\prime }}{k_{L}S_{L}}\right)}^{2}+{\left(\frac{{∆C}^{\prime }}{k_{C}S_{C}}\right)}^{2}+{\left(\frac{{∆H}^{\prime }}{k_{H}S_{H}}\right)}^{2}+R_{T}\left(\frac{{∆C}^{\prime }}{k_{C}S_{C}}\right)\left(\frac{{∆H}^{\prime }}{k_{H}S_{H}}\right)}$$

The values for each stage of analysis were obtained by subtracting the baseline values from those obtained after T1, T2 and T3. The thresholds of acceptability and perceptibility of the color change were determined using the parameters described by Ghinea et al. [28], with the color change being clinically acceptable when ΔE00 = 2.23 and perceptible when ΔE00 = 1.25.

### 2.7. Surface Roughness Assessment

Surface roughness was measured using a Confocal Laser Scanning Microscope (CLSM) (LEXT OLS4000, Olympus, Breinigsville, PA, USA), using a 5× objective lens in the center of the sample. A topographic image of the surface was obtained and then the average roughness (Sa), in µm, was measured using the proprietary software for the confocal microscope OLS4000that provided the values. All measurements were taken by the same calibrated operator.

### 2.8. Knoop Microhardness Evaluation

The microhardness analysis was carried out using the HMV-2 series microhardness meter (Shimadzu Corp., Kyoto, Japan). Five dents were made in each sample using a Knoop penetrator (Shimadzu Corp.) and a load of 25 g (245 mN) for 5 s. The diagonals of the dents were measured by the device itself, which provided the results at the end of the 5 measurements. The arithmetic mean of the readings was taken to obtain the Knoop microhardness (KHN). All measurements were taken by the same calibrated operator.

### 2.9. Statistical Analysis

The color change (∆E_00_), microhardness, and surface roughness of the specimens were evaluated at four experimental times: T1—Baseline, T2—After 1st Immersion, T3—After Brushing, T4—After 2nd Immersion. For the statistical analysis, deltas between consecutive times were calculated to measure the effective change at each stage of the protocol: ∆1 (T1-T2), ∆2 (T2-T3), and ∆3 (T3-T4) (Figure 1). Negative delta values indicate an increase, while positive delta values indicate a decrease in the evaluated parameters. The data were tabulated, presented as mean ± standard deviation, and subjected to statistical analysis using IBM SPSS 20.0 for Windows (SPSS Inc., Chicago, IL, USA). The Shapiro–Wilk test confirmed normal distribution of the data, so Repeated Measures ANOVA followed by Bonferroni’s post hoc test was applied. The α used for the comparisons between groups was 5% (*p* < 0.05).

## 3. Results

All original data was provided as Supplementary Materials.

### 3.1. Color Change

**Figure 4:**
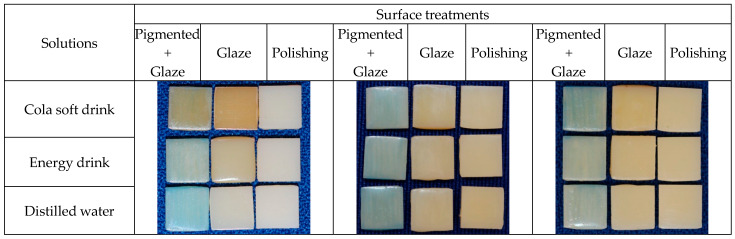
PrintaX samples: After 1st immersion; After 2nd immersion; and After brushing.

**Figure 5:**
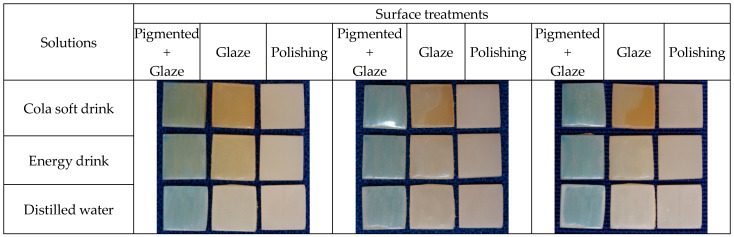
Nanolab samples: After 1st immersion; After 2nd immersion; and After brushing.

**Figure 6:**
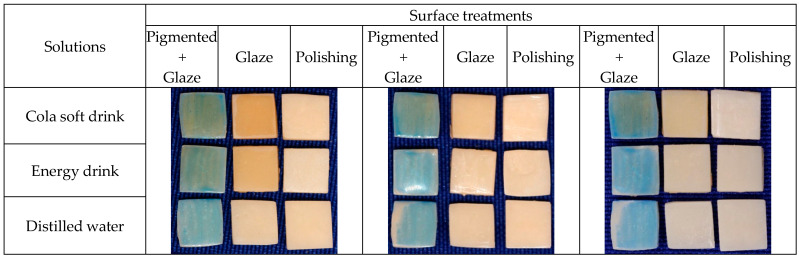
Duralay samples: After 1st immersion; After 2nd immersion; and After brushing.

The color change (∆E_00_) of the specimens was measured at each stage of the study: T1—Baseline, T2—After 1st Immersion, T3—After Brushing, T4—After 2nd Immersion. Deltas between consecutive times (∆1: T1-T2, ∆2: T2-T3, ∆3: T3-T4) were calculated as described in the Statistical Analysis section. For ∆E_00_, resin (*p* < 0.05), treatments (*p* < 0.05) and solutions (*p* < 0.05) significantly influenced the results. In addition, the interactions resin*treatment (*p* < 0.05), resin*solution (*p* < 0.05), treatment*solution (*p* < 0.05) and resin*treatment*solution (*p* < 0.05) were significant. Figure 4, Figure 5 and Figure 6 and Table 3 show the detailed data for the color variation values (∆E_00_) for each resin, treatment and solution.

Analysis of color change revealed significant differences between materials and solutions. Nanolab showed the greatest color change in Cola soft drink (ΔE_00_ = 4.2 ± 0.3), a clinically perceptible value (ΔE > 3.5). PrintaX demonstrated the best color stability, with ΔE_00_ values below the clinical threshold in all solutions (Cola soft drink: 2.8 ± 0.2; Energy drink: 2.5 ± 0.2). Duralay showed intermediate values (Cola soft drink: 3.5 ± 0.3; Energy drink: 3.2 ± 0.2). Distilled water caused minimal changes in all materials (ΔE_00_ < 1.5), confirming that the changes are attributable to the acidity of the beverages. Nanolab showed significantly greater color change than PrintaX in Cola soft drink (4.2 vs. 2.8, *p* < 0.01). There was no significant difference in color change between Cola soft drink and Energy drink for Duralay (3.5 vs. 3.2, *p* > 0.05).

In general, the analyses in Table 3 show that:

- Nanolab is significantly more susceptible to staining at ∆1 (1st immersion), especially with Cola soft drink and Energy Drink in treatments with Extrinsic pigment + Glaze and Glaze;
- Duralay and PrintaX show more similar behavior, with less initial staining;
- At ∆3 (2nd immersion), the differences between resins decrease considerably;
- Nanolab shows a drastic reduction in ∆E from ∆1 to ∆3 in practically all scenarios (pattern A → B → C = highly significant);
- Duralay with Extrinsic pigment + Glaze and Glaze also shows a significant reduction (A → B → C);
- PrintaX with Polishing shows temporal stability (A → A → A), indicating less color variation over time.

It is possible to note that Nanolab resin is significantly more susceptible to initial staining (∆1), especially with acidic drinks; Polishing is the most effective treatment in reducing staining over time; differences between resins are more pronounced in ∆1 and ∆2, converging in ∆3; and Cola soft drink causes more staining than Energy Drink in virtually all scenarios.

### 3.2. Surface Roughness

Surface roughness was evaluated at T1-T4, with deltas between consecutive times (∆1-∆3) calculated as described in Section 2.9. For surface roughness, all the variables were significant, treatments (*p* < 0.05), resins (*p* = 0.003) and solutions (*p* = 0.007), as well as the interactions treatment*resin, treatment*solution, resin*solution and resin*treatment*solution (*p* < 0.05). The mean roughness values and standard deviation are shown in Table 4.

**Figure 7:**
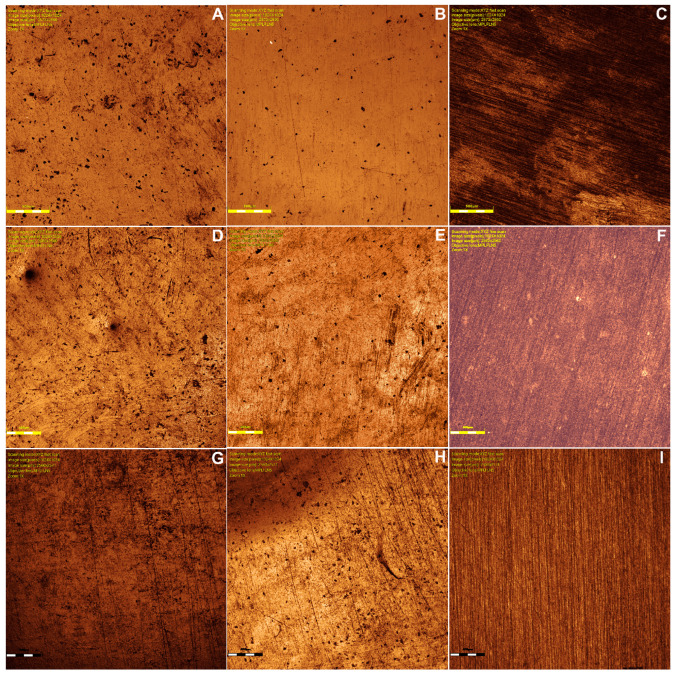
Confocal images of: (**A**) pigmented/glazed PrintaX sample; (**B**) glazed PrintaX sample; (**C**) polished PrintaX sample; (**D**) pigmented/glazed Nanolab sample; (**E**) glazed Nanolab sample; (**F**) Polished Nanolab sample; (**G**) pigmented/glazed Duralay sample; (**H**) glazed Duralay sample; and (**I**) polished Duralay sample.

Confocal microscope images are shown in Figure 7A–I for each resin/treatment.

Confocal microscopy images and measurement results showed that Nanolab exhibited the highest roughness in cola soft drink (0.48 ± 0.05 μm), significantly higher than PrintaX (0.32 ± 0.03 μm, *p* < 0.05). The energy drink caused an intermediate increase in roughness (Nanolab: 0.42 ± 0.04 μm; PrintaX: 0.28 ± 0.03 μm). Duralay showed similar roughness to Nanolab in cola soft drink (0.38 ± 0.04 μm). The control group (distilled water) maintained roughness below 0.25 μm for all materials. PrintaX maintained significantly lower roughness change than Nanolab after immersion in energy drink (0.28 vs. 0.42 μm, *p* < 0.05).

Nanolab exhibited significantly higher roughness than PrintaX in both Cola soft drink (*p* < 0.01) and Energy drink (*p* < 0.05)

Cola soft drink produced significantly greater roughness than Energy drink for all materials (*p* < 0.05)

All materials in acidic solutions showed roughness values significantly above the 0.2 μm clinical threshold (*p* < 0.001)

### 3.3. Knoop Microhardness

Knoop microhardness was assessed at T1-T4, and deltas between consecutive times (∆1-∆3) were calculated as described in the Statistical Analysis section. The average KHN values are shown in Table 5. The statistical analyses indicated that resin (*p* = 0.023), treatment (*p* < 0.05) and solution (*p* = 0.013), as well as the interactions resin*treatment (*p* < 0.05), resin*solution (*p* < 0.05), treatment*solution (*p* < 0.05), and resin*treatment*solution (*p* < 0.012) significantly influenced the KHN results.

All materials experienced a significant reduction in hardness after immersion in acidic solutions. Nanolab showed the greatest percentage reduction in Cola soft drink (24.3%), followed by Duralay (19.8%) and PrintaX (15.2%). Energy drink caused similar reductions (20–22% for Nanolab and Duralay, 13% for PrintaX). The reduction in hardness was significantly greater for Nanolab compared to PrintaX in Cola soft drink (24.3% vs. 15.2%, *p* < 0.05). The Cola soft drink caused significantly greater hardness reduction than Energy drink for Nanolab (*p* < 0.05) and Duralay (*p* < 0.05).

In the microhardness results, it is possible to observe that the PrintaX resin consistently demonstrated the highest Knoop microhardness values compared to the Duralay and Nanolab resins in almost all conditions evaluated. This performance was particularly noteworthy when PrintaX was subjected to the Polishing treatment, where it reached peak surface hardness.

Immersion in the soft drink cola solution was the most aggressive condition for the surface integrity of the resins. In all resins and treatments, exposure to soft drink cola resulted in significant drops in hardness over times ∆1, ∆2, and ∆3, indicating progressive surface degradation.

Unlike the Glaze and Extrinsic pigment + Glaze treatments, Polishing appeared to confer greater resistance or even a slight gain in hardness under some conditions. Although soft drink cola still caused degradation, polishing maintained PrintaX at a higher hardness level, suggesting that this treatment can optimize surface mechanical properties.

The Extrinsic Pigment + Glaze treatment, when combined with immersion in Energy drink, proved to be one of the most stable conditions. Under this combination, the resins showed little or no significant change in hardness between times ∆1 and ∆3, indicating good maintenance of surface integrity throughout the evaluation period.

These results show variations in resin responses to different treatments and solutions, highlighting the complexity of the interactions between the factors analyzed.

## 4. Discussion

The null hypothesis tested in this study posited that there would be no changes in the optical and mechanical properties of the analyzed resins, regardless of resin type, surface finishing, or immersion solution. However, analysis of the results led to the rejection of this hypothesis due to significant differences observed in all evaluated properties. These findings indicate that surface finishing, immersion in solutions, and brushing can significantly influence the optical and mechanical characteristics of provisional 3D-printed resins.

In this study, all resins exhibited ∆E values exceeding the clinically acceptable threshold (∆E_00_ ≤ 2.23) [28] after the first immersion, with Nanolab demonstrating the most pronounced changes. A decrease in these values was observed following the brushing test, suggesting that brushing effectively removes surface pigments. Notably, Nanolab with extrinsic pigment + glaze treatment, when immersed in water, was the only sample to exhibit a ∆E_00_ value within the clinically perceptible threshold (∆E_00_ ≤ 1.25) [28]. Clinically acceptable values were recorded for Duralay polished and immersed in cola soft drink (∆E_00_ = 2.23), PrintaX with glaze immersed in water (∆E = 1.56), and PrintaX polished across all solutions (∆E ≤ 2.23).

After the second immersion, only polished Nanolab immersed in a cola beverage remained within the clinically perceptible threshold (∆E_00_ = 1.16). These results align with the findings of Peñate et al. [14], who reported that samples receiving a glaze finish exceeded clinical acceptability parameters.

Following the second immersion, some glazed samples exhibited detachment and surface cracking, which facilitated the deposition of colorants in these regions. This observation is consistent with the study by Raszewski, Chojnacka, and Mikulewicz [20], which reported similar findings but also concluded that, despite material degradation over time, glazed samples experienced greater color changes compared to polished and unpolished surfaces. Discoloration in resin restorative materials is often attributed to the oxidation of the polymer matrix or unreacted double bonds from residual monomers [10,14]. Although printed resins undergo post-curing, their polymerization rates remain relatively low, leading to compromised surface integrity and increased susceptibility to discoloration and deterioration due to residual monomers [8,12,29].

In this study, printed resins exhibited less color change than conventional resins. Research by Shin et al. [10] and Taşın et al. [12] has established a correlation between prolonged immersion time and increased color change. This may explain the significant ∆E values observed after the first immersion, as color measurements were taken only after six days rather than at shorter intervals. Among the tested solutions, cola soft drink induced the most significant color change, particularly in the pigment + glaze and glaze-only groups, followed by energy drink and distilled water.

After the brushing test, ∆E_00_ values decreased, reinforcing the notion that brushing aids in pigment removal, consistent with the findings of Peñate et al. [14]. This difference was also visually perceptible, as samples finished with Blue pigment displayed a greenish hue following immersion, likely due to interactions between the caramel dye in cola soft drink and the pigment on the sample surface. At the time of writing, no other studies have been found that specifically examine the application of pigment to samples; existing literature primarily focuses on glaze applications.

Several factors may contribute to the color changes observed, including variations in material composition among manufacturers [30], insufficient curing of the final resin layer [31], absence of filler particles [3], and differences in monomer conversion and polarity [9]. It is important to note that the exact chemical composition of 3D printing materials for provisional restorations remains undisclosed by manufacturers [11].

Assessing color changes in terms of perceptibility and acceptability is clinically significant, particularly for anterior restorations. The results of this study indicate that immersion in solutions and the type of surface finishing substantially impact material coloration. Over time, these changes can become clinically unacceptable, leading to patient dissatisfaction.

In terms of surface roughness, Nanolab exhibited the highest average values, followed by Duralay and PrintaX. The pigment + glaze finishing ranged from −0.23 µm to 0.33 µm, glaze-only ranged from −0.19 µm to 0.54 µm, and polished ranged from −1.55 µm to 0.13 µm. Only PrintaX with pigment + glaze and glaze-only treatments exceeded the plaque accumulation threshold of 0.2 µm but remained below the clinically unacceptable threshold of 10 µm, suggesting their stability for medium- to long-term use [12].

Jain et al. [3] and Ellakany et al. [19] reported that applying light-curing surface sealants increased surface roughness, while Taşın et al. [12] observed the opposite effect. The present study did not identify a consistent pattern in roughness changes after brushing. While Duralay showed a decreased roughness in the pigment + glaze group, Nanolab exhibited an increase in this same category. PrintaX demonstrated a decrease in roughness in both the pigment + glaze and polished groups, suggesting that the interaction between resin type and finishing method may yield non-linear results.

A previous study comparing resin roughness based on manufacturing methods [32] concluded that milled resins exhibit the lowest roughness values compared to printed and conventional resins. The layer-by-layer buildup in printed resins may result in greater porosity, whereas conventional resins, mixed manually, can trap air bubbles during processing. Conversely, milled resins are derived from pre-polymerized blocks manufactured under high pressure and temperature, which enhances their surface properties [10,13,19,30].

Microhardness (KHN) values were highest for Duralay, followed by Nanolab and PrintaX. The pigment + glaze group ranged from −1.08 to 0.18, the glaze-only group ranged from −0.35 to −0.75, and the polished group ranged from −0.92 to −2.17. These results suggest that conventional resins exhibit higher microhardness than printed resins, contrasting with previous findings that reported the opposite [33]. Following the brushing test, all resins experienced a decline in microhardness, likely due to the abrasive effects of toothpaste. However, after the second immersion, an increase was observed for Nanolab with pigment + glaze and for PrintaX with polish.

After the second immersion, most groups exhibited reduced microhardness, except for Nanolab with pigment + glaze and PrintaX with polish. Alageel et al. [11] suggested that thermocycling alters resin physical properties, facilitating water molecule penetration and leading to resin expansion, polymer matrix rupture, and microhardness reduction. Oh et al. [34] observed a similar reduction in microhardness in printed resins exposed to washing liquid temperatures exceeding 30 °C for varying durations. The increased temperature weakened the polymer network, promoting chemical elution and subsequent microhardness loss [3].

Both Alageel et al. [11] and Oh et al. [34] support the findings of this study, offering plausible explanations for the observed microhardness reductions. Although thermocycling was not conducted, immersion in solutions at 37 °C likely contributed to similar outcomes. Additionally, the immersion duration in this study was substantially longer than that used by Oh et al. [34], potentially amplifying the observed effects.

The color changes observed have significant clinical implications. The findings confirm that immersion in solutions and surface finishing methods substantially influence the coloration of tested materials, potentially leading to patient dissatisfaction over time. Clinically, chemical degradation of provisional resins may simultaneously compromise aesthetics and maintenance. Higher ∆E values indicate a greater risk of perceptible discoloration over time, whereas increased surface roughness (Ra) promotes biofilm retention and staining, making hygiene more challenging. Reduced Knoop microhardness (KHN) suggests higher susceptibility to wear and surface damage during brushing and function, potentially accelerating gloss loss and aesthetic deterioration. Therefore, materials showing lower ∆E, smaller Ra increase, and reduced KHN loss may be more suitable for provisional restorations under acidic challenges and routine oral hygiene.

This in vitro comparative study has limitations. The flat shape of the samples may not fully replicate the clinical conditions of real restorations, and the study was conducted entirely in a laboratory setting, excluding factors such as occlusal forces and saliva. Similarly, the solutions used, although widely consumed by the general population, do not represent the full range of available options. A more in-depth analysis of results based on material composition was not feasible, as the formulations of new 3D-printed resins remain undisclosed by manufacturers. This lack of transparency limits the ability to establish correlations regarding monomer composition, photoinitiators, and filler particles.

However, controlled conditions during the experiments allowed for a standardized comparison of different materials, surface finishes, immersion solutions, and testing procedures.

3D resins hold promise for provisional dental rehabilitation, yet standardized protocols for these materials remain lacking in the literature. Future studies should explore the effects of aging and occlusal forces while conducting more comprehensive analyses of resin properties. Further research is crucial for developing guidelines that enhance the clinical performance of 3D resins, prolonging their intraoral durability and optimizing their physical and mechanical characteristics.

## 5. Conclusions

Within the limitations of this study, acidic challenge promoted significant changes in the optical and surface properties of the evaluated resins, increasing ∆E and roughness and reducing microhardness to different extents depending on the material. Clinically, these findings highlight the relevance of material selection and limiting exposure to acidic beverages during provisional use.

## Figures and Tables

**Table 1:** Materials used in the study.

Material	Composition	Indication	Manufacturer
PrintaX AA Temp	Aromatic methacrylic oligomer (<80%), aliphatic methacrylic oligomer (<30%), phosphine oxide (<5%)	3D Printing	Odontomega, Brazil
Nanolab 3D	Nanohybrid resin (1419, 210,102) Proprietary	3D Printing	Wilcos, Brazil
Duralay	Powder: benzoyl peroxide (0–10%), dialkyl phthalate (0–20%), residual monomers, titanium dioxide, mineral pigment, disazo pigment Liquid: methyl methacrylate (90–100%)	Conventional	Reliance Dental Mfg., LLC. Worth, IL, USA

**Table 2:** Printing parameters for each resin.

Printing Parameters								
	Layer Height (mm)	Base Layers	Exposure Time (s)	Base Exposure Time (s)	Lift Speed (mm/min)	Retraction Speed (mm/min)	Lifting Distance (mm)	Retraction Distance (mm)
PrintaX	0.05	10	4	40	65	100	5	5
Nanolab	0.05	10	3.6	40	65	100	5	5

**Table 3:** Color change (∆E_00_) of all resins, treatments, solutions and times.

Color Change (∆E_00_)					
			∆1	∆2	∆3
Resins	Treatments	Solutions	Mean ± SD	Mean ± SD	Mean ± SD
Duralay	Extrinsic Pigment + Glaze	Cola soft drink	13.12 ± 1.90 ^aA^	9.71 ± 2.40 ^aB^	3.57 ± 2.03 ^aC^
		Energy drink	8.86 ± 0.82 ^aA^	7.86 ± 1.24 ^aA^	1.64 ± 1.01 ^aB^
		Distilled Water	1.91 ± 0.55 ^aA^	4.66 ± 2.87 ^aB^	4.11 ± 2.93 ^aB^
	Glaze	Cola soft drink	13.36 ± 3.19 ^aA^	9.21 ± 3.23 ^aB^	2.62 ± 2.73 ^aC^
		Energy drink	7.84 ± 0.83 ^aA^	6.17 ± 1.03 ^aA^	1.80 ± 0.54 ^aB^
		Distilled Water	1.62 ± 0.86 ^aA^	2.30 ± 1.37 ^aA^	1.62 ± 0.89 ^aA^
	Polishing	Cola soft drink	2.93 ± 3.36 ^aA^	2.5 ± 1.68 ^aA^	1.99 ± 1.76 ^aA^
		Energy drink	2.12 ± 2.04 ^aA^	4.00 ± 2,85 ^aB^	3.91 ± 3.50 ^aB^
		Distilled Water	1.35 ± 0.77 ^aA^	2.55 ± 1.69 ^aA^	1.98 ± 1.04 ^aA^
Nanolab	Extrinsic Pigment + Glaze	Cola soft drink	20.06 ± 1.29 ^bA^	12.4 ± 2.81 ^bB^	2.86 ± 0.68 ^aC^
		Energy drink	16.04 ± 0.79 ^bA^	8.64 ± 2.63 ^aB^	3.79 ± 2.51 ^bC^
		Distilled Water	12.38 ± 0.52 ^bA^	1.73 ± 0.66 ^bB^	4.48 ± 0.39 ^aC^
	Glaze	Cola soft drink	22.21 ± 3.13 ^bA^	9.00 ± 1.99 ^aB^	1.94 ± 1.02 ^abC^
		Energy drink	17.28 ± 0.82 ^bA^	6.85 ± 3.19 ^aB^	3.27 ± 1.85 ^aC^
		Distilled Water	12.11 ± 0.43 ^bA^	4.73 ± 1.20 ^bB^	8.36 ± 1.30 ^bC^
	Polishing	Cola soft drink	17.41 ± 0.81 ^bA^	3.10 ± 1.23 ^aB^	1.16 ± 0.68 ^abC^
		Energy drink	16.00 ± 0.99 ^bA^	3.63 ± 1.06 ^aB^	1.79 ± 0.68 ^bC^
		Distilled Water	11.93 ± 1.70 ^bA^	8.85 ± 1.96 ^bB^	10.93 ± 1.17 ^bA^
PrintaX	Extrinsic Pigment + Glaze	Cola soft drink	14.38 ± 3.46 ^aA^	14.29 ± 1.94 ^bA^	3.15 ± 1.55 ^aB^
		Energy drink	7.64 ± 2.01 ^aA^	6.85 ± 1.22 ^aA^	3.32 ±1.82 ^abB^
		Distilled Water	4.12 ± 1.19 ^cA^	1.28 ± 0.73 ^bB^	1,.0 ± 0.68 ^bB^
	Glaze	Cola soft drink	10.40 ± 1.27 ^cA^	11.44 ± 2.16 ^bA^	4.03 ± 2.03 ^aB^
		Energy drink	5.00 ± 0.80 ^cA^	7.37 ± 1.41 ^aB^	3.17 ± 1.47 ^aC^
		Distilled Water	5.13 ± 0.75 ^cA^	1.56 ± 1.18 ^aB^	1.99 ± 1.18 ^aB^
	Polishing	Cola soft drink	2.29 ± 0.87 ^aA^	1.46 ± 0.83 ^aAB^	3.16 ± 1.72 ^aA^
		Energy drink	2.63 ± 1.12 ^aA^	1.58 ± 0.62 ^bA^	2.27 ± 1.49 ^abA^
		Distilled Water	2.47 ± 0.81 ^aA^	1.37 ± 0.59 ^aA^	2.14 ± 0.97 ^aA^

Data are expressed as Mean ± SD. Lowercase letters (a, b, c) indicate comparisons between resins within the same column (i.e., for the same treatment, and immersion solution). Groups sharing the same lowercase letter showed no significant difference (*p* > 0.05), while groups with different lowercase letters showed a significant difference (*p* < 0.05). Uppercase letters (A, B, C) indicate comparisons between times (Δ1, Δ2, Δ3) within the same row (i.e., for the same resin, treatment, and immersion solution). Time groups sharing the same uppercase letter showed no significant difference (*p* > 0.05), while groups with different uppercase letters showed a significant difference (*p* < 0.05).

**Table 4:** Surface roughness for all resins, treatments, solutions and times.

Surface Roughness—Sa (µm)					
			∆1	∆2	∆3
Resins	Treatments	Solutions	Mean ± SD	Mean ± SD	Mean ± SD
Duralay	Extrinsic pigment + Glaze	Cola soft drink	0.64 ± 0.46 ^aA^	−0.06 ± 0.75 ^aA^	−0.03 ± 0.19 ^aA^
		Energy drink	0.50 ± 1.29 ^aA^	0.44 ± 0.45 ^aA^	−0.18 ± 0.37 ^aA^
		Distilled Water	0.82 ± 1.36 ^aA^	0.36 ± 0.66 ^aA^	−0.34 ± 0.68 ^aA^
	Glaze	Cola soft drink	0.78 ± 0.76 ^aA^	0.34 ± 0.47 ^aA^	−0.25 ±0.22 ^aA^
		Energy drink	−0.19 ± 0.5 ^aA^	0.52 ± 0.49 ^aA^	−0.07 ± 0.44 ^aA^
		Distilled Water	0.66 ± 0.46 ^aA^	0.0051 ± 0.36 ^aA^	−0.06 ± 0.25 ^aA^
	Polishing	Cola soft drink	−0.11 ± 0.24 ^aA^	0.011 ± 0.65 ^aA^	−0.12 ± 0.45 ^aA^
		Energy drink	−0.06 ± 0.52 ^aA^	−0.71 ± 1.6 ^aA^	−0.15 ± 0.45 ^abA^
		Distilled Water	−0.05 ± 0.34 ^aA^	0.16 ± 0.44 ^aA^	−0.18 ± 0.38 ^aA^
Nanolab	Extrinsic pigment + Glaze	Cola soft drink	0.23 ± 0.40 ^aA^	0.60 ± 0.80 ^aA^	−0.08 ± 0.11 ^aA^
		Energy drink	0.35 ± 0.51 ^aA^	0.68 ± 0.98 ^aA^	0.02 ± 0.19 ^aA^
		Distilled Water	0.14 ± 0.37 ^aA^	0.0053 ± 0.52 ^aA^	0.006 ± 0.25 ^aA^
	Glaze	Cola soft drink	0.13 ± 0.11 ^aA^	−0.37 ± 0.34 ^aA^	−0.14 ± 0.30 ^aA^
		Energy drink	−0.02 ± 0.14 ^aA^	−0.28 ± 0.5 ^aA^	0.06 ± 0.29 ^aA^
		Distilled Water	0.04 ± 0.23 ^aA^	−0.48 ± 0.44 ^aA^	0.08 ± 0.15 ^aA^
	Polishing	Cola soft drink	0.35 ± 0.25 ^aA^	−0.45 ± 0.46 ^aA^	0.04 ± 0.26 ^aA^
		Energy drink	0.09 ± 0.26 ^abA^	−0.40 ± 0.24 ^aA^	0.07 ± 0.22 ^aA^
		Distilled Water	0.16 ± 0.14 ^aA^	−0.49 ± 0.16 ^aA^	0.25 ± 0.15 ^bA^
PrintaX	Extrinsic pigment + Glaze	Cola soft drink	−0.57 ± 0.82 ^aA^	0.61 ± 0.59 ^aA^	−0.77 ± 0.42 ^bA^
		Energy drink	−1.07 ± 1.80 ^aA^	0.83 ± 1.74 ^aA^	−0.87 ± 0.57 ^bAB^
		Distilled Water	0.09 ± 0.34 ^aA^	−0.38 ± 0.28 ^aA^	−0.90 ± 0.45 ^bA^
	Glaze	Cola soft drink	−0.93 ± 2.53 ^aA^	0.11 ± 2.25 ^aA^	−0.79 ± 0.38 ^bA^
		Energy drink	−0.34 ± 0.26 ^aA^	−0.62 ± 0.29 ^aA^	−0.67 ± 0.59 ^bA^
		Distilled Water	−0.17 ± 0.28 ^aA^	−0.93 ± 0.3 ^aA^	−0.51 ± 0.38 ^bA^
	Polishing	Cola soft drink	−14.99 ± 5.05 ^bA^	17.51 ± 5.62 ^bB^	−0.89 ± 0.57 ^bC^
		Energy drink	2.30 ± 6.81 ^bA^	3.11 ± 5.69 ^bA^	−0.57 ± 0.77 ^bB^
		Distilled Water	3.63 ± 5.26 ^bA^	4.05 ± 2.11 ^bA^	0.21 ± 0.28 ^aB^

Data are expressed as Mean ± SD. Lowercase letters (a, b, c) indicate comparisons between resins within the same column (i.e., for the same treatment and immersion solution). Groups sharing the same lowercase letter showed no significant difference (*p* > 0.05), while groups with different lowercase letters showed a significant difference (*p* < 0.05). Uppercase letters (A, B, C) indicate comparisons between times (Δ1, Δ2, Δ3) within the same row (i.e., for the same resin, treatment, and immersion solution). Time groups sharing the same uppercase letter showed no significant difference (*p* > 0.05), while groups with different uppercase letters showed a significant difference (*p* < 0.05).

**Table 5:** Knoop microhardness for all resins, treatments, solutions and times.

Knoop Microhardness (KHN)					
			∆1	∆2	∆3
Resin	Treatments	Solutions	Mean ± SD	Mean ± SD	Mean ± SD
Duralay	Extrinsic pigment + Glaze	Cola soft drink	1.84 ± 0.36 ^abA^	0.91 ± 0.69 ^abAB^	−0.47 ± 0.45 ^aB^
		Energy drink	1.17 ± 0.95 ^aA^	1.36 ± 0.85 ^aA^	−0.33 ± 0.60 ^aA^
		Distilled Water	−0.37 ± 0.83 ^abA^	2.5 ± 0.91 ^aB^	−0.65 ± 0.53 ^abA^
	Glaze	Cola soft drink	1.40 ± 0.45 ^aA^	0.81 ± 0.48 ^aAB^	−0.59 ± 0.34 ^aB^
		Energy drink	1.21 ± 0.66 ^aA^	1.99 ± 0.66 ^aA^	−0.97 ± 0.8 ^abB^
		Distilled Water	−0.05 ± 1.13 ^abA^	2.56 ± 1.28 ^aB^	−0.39 ± 0.58 ^aA^
	Polishing	Cola soft drink	−0.09 ± 0.78 ^aA^	3.21 ± 0.67 ^aB^	−0.15 ± 0.76 ^aA^
		Energy drink	−1.01 ± 0.63 ^aA^	3.33 ± 0.89 ^aB^	0.06 ± 0.54 ^aA^
		Distilled Water	−0.44 ± 0.67 ^aA^	3.49 ± 0.56 ^aB^	−0.07 ± 0.83 ^aA^
Nanolab	Extrinsic pigment + Glaze	Cola soft drink	−0.24 ± 1.53 ^aA^	−0.30 ± 2.29 ^aA^	−0.22 ± 1.92 ^aA^
		Energy drink	−2.60 ± 2.01 ^bA^	2.33 ± 1.73 ^aB^	−0.18 ± 0.88 ^aC^
		Distilled Water	−2.26 ± 1.77 ^aA^	3.45 ± 1.41 ^aB^	−1.62 ± 0.70 ^aA^
	Glaze	Cola soft drink	0.54 ± 1.61 ^abAB^	1.74 ± 1.17 ^aA^	−1.12 ± 0.75 ^aB^
		Energy drink	0.003 ± 2.11 ^aA^	3.17 ± 0.93 ^aB^	−0.73 ± 0.81 ^aA^
		Distilled Water	0.61 ± 2.85 ^aA^	3.27 ± 1.43 ^aB^	−0.70 ± 1.09 ^aA^
	Polishing	Cola soft drink	1.62 ± 3.17 ^abA^	3.39 ± 1.56 ^aA^	1.53 ± 1.07 ^bA^
		Energy drink	1.44 ± 2.71 ^bA^	2.13 ± 1.07 ^aA^	2.68 ± 0.77 ^bA^
		Distilled Water	2.60 ± 3.13 ^bAB^	3.27 ± 1.23 ^aA^	0.86 ± 0.54 ^aB^
PrintaX	Extrinsic pigment + Glaze	Cola soft drink	3.87 ± 2.70 ^bA^	2.43 ± 2.26 ^bA^	−0.14 ± 0.92 ^aB^
		Energy drink	0.86 ± 2.29 ^aA^	2.37 ± 2.15 ^aA^	−2.34 ± 2.13 ^bB^
		Distilled Water	0.84 ± 1.04 ^bAB^	1.89 ± 1.44 ^aA^	−0.07 ± 1.28 ^bB^
	Glaze	Cola soft drink	−1.20 ± 2.73 ^bA^	1.32 ± 3.58 ^aB^	−0.12 ± 1.6 ^aA^
		Energy drink	1.27 ± 3.30 ^aA^	2.54 ± 1.75 ^aA^	−2.32 ± 2.34 ^bB^
		Distilled Water	−1.82 ± 2.48 ^bA^	3.03 ± 2.33 ^aB^	0.53 ± 2.77 ^aC^
	Polishing	Cola soft drink	2.59 ± 2.41 ^bA^	−0.78 ± 2.32 ^bB^	5.30 ± 1.89 ^cC^
		Energy drink	−1.42 ± 2.35 ^aA^	−1.97 ± 1.5 ^bA^	5.81 ± 2.49 ^cB^
		Distilled Water	−1.96 ± 2.28 ^aA^	2.28 ± 2 ^aB^	4.34 ± 2.44 ^bC^

Data are expressed as Mean ± SD. Lowercase letters (a, b, c) indicate comparisons between resins within the same column (i.e., for the same treatment and immersion solution). Groups sharing the same lowercase letter showed no significant difference (*p* > 0.05), while groups with different lowercase letters showed a significant difference (*p* < 0.05). Uppercase letters (A, B, C) indicate comparisons between times (Δ1, Δ2, Δ3) within the same row (i.e., for the same resin, treatment, and immersion solution). Time groups sharing the same uppercase letter showed no significant difference (*p* > 0.05), while groups with different uppercase letters showed a significant difference (*p* < 0.05).

## Data Availability

All original data were available in Supplementary Material.

## Supplementary Materials

The following supporting information (original data) can be downloaded at: https://www.mdpi.com/article/10.3390/medicina62020382/s1. File S1: Color Change; File S2: Knoop Microhardness; File S3: Roughness.
