# Supplementary material for: 3D-Printed Versus Conventional Dental Provisional Resins: A Comparative Study
Source: Medicina (Kaunas). 2026 Feb 14;62(2):382. doi: 10.3390/medicina62020382 (PMC12942832; doi:10.3390/medicina62020382)
Supplement: Supplementary file 1 [file medicina-62-00382-s001.zip › S1_Color Change.pdf]

Color Change

1-10: Immersed in cola soft drink
11-20: Immersed in energy drink
21-30: Immersed in distilled water

Δ1: T0-T1 (Initial - After 1st Immersion)
Δ2: T1-T2 (After 1st Immersion - After Brushing)
Δ2: T1-T3 (After Brushing - After 2nd Immersion)

Resin	Sample	Subgroups	Δ1	Δ2	Δ3
Printax	1	PG	11,9529	14,5432	2,6161
Printax	2	PG	12,2856	13,0892	1,7917
Printax	3	PG	14,9287	13,0384	2,6005
Printax	4	PG	14,5304	16,4555	3,5983
Printax	5	PG	12,2270	15,4491	3,3194
Printax	6	PG	9,5015	15,9103	5,2119
Printax	7	PG	13,0735	13,0915	3,3427
Printax	8	PG	17,2440	11,8306	2,1301
Printax	9	PG	16,3220	17,4673	6,0656
Printax	10	PG	21,8311	12,1207	0,8682
Printax	11	PG	12,7640	5,8555	1,2431
Printax	12	PG	6,4042	6,2712	2,6758
Printax	13	PG	5,2156	8,0796	2,4369
Printax	14	PG	6,9698	6,7210	2,3812
Printax	15	PG	7,9479	8,0081	4,3132
Printax	16	PG	7,3193	4,1432	1,0617
Printax	17	PG	7,6079	7,7824	4,6532
Printax	18	PG	6,5909	7,8155	2,7690
Printax	19	PG	7,0503	6,7564	4,7503
Printax	20	PG	8,5515	7,1526	6,9603
Printax	21	PG	5,5083	1,0275	0,7918
Printax	22	PG	6,0588	3,0829	1,7759
Printax	23	PG	5,6736	0,4960	1,8287
Printax	24	PG	3,4346	1,2940	1,8252

Resin	Sample	Subgroups	Δ1	Δ2	Δ3
Nanolab	1	PG	22,2383	15,6311	3,3013
Nanolab	2	PG	20,0143	10,5617	2,6580
Nanolab	3	PG	20,3766	11,4049	3,1059
Nanolab	4	PG	20,2870	18,3339	3,3159
Nanolab	5	PG	20,7118	13,1784	2,7531
Nanolab	6	PG	17,3569	11,0659	3,1478
Nanolab	7	PG	20,7836	10,2393	1,3769
Nanolab	8	PG	20,3776	13,7487	2,0740
Nanolab	9	PG	18,7143	9,3850	3,4161
Nanolab	10	PG	19,8104	10,5418	3,5221
Nanolab	11	PG	16,5222	7,5058	3,3648
Nanolab	12	PG	15,7926	7,9575	3,2237
Nanolab	13	PG	16,3661	7,5116	2,1288
Nanolab	14	PG	16,6112	7,8512	3,2787
Nanolab	15	PG	14,7455	7,9149	3,2127
Nanolab	16	PG	17,3057	8,1924	3,3112
Nanolab	17	PG	15,4272	16,0169	10,8080
Nanolab	18	PG	16,3415	8,6063	3,5884
Nanolab	19	PG	16,3176	8,0116	2,7252
Nanolab	20	PG	14,9841	6,8603	2,2702
Nanolab	21	PG	12,0241	1,7229	4,0792
Nanolab	22	PG	12,8625	1,6778	4,4167
Nanolab	23	PG	12,6301	0,0197	4,6646
Nanolab	24	PG	13,1020	2,4594	4,4253

Printax	25	PG	3,5555	1,8110	1,4160
Printax	26	PG	3,8667	1,1695	0,8445
Printax	27	PG	2,6692	0,8977	1,4182
Printax	28	PG	4,1287	0,6308	0,6301
Printax	29	PG	3,3542	1,0440	0,7421
Printax	30	PG	3,0167	1,3718	2,8142
Printax	1	G	9,1354	13,0008	1,9988
Printax	2	G	11,5693	12,6829	6,1072
Printax	3	G	12,9320	12,3067	2,5368
Printax	4	G	11,1661	10,2258	2,0047
Printax	5	G	9,9049	13,3289	5,2872
Printax	6	G	9,4121	6,5681	1,7142
Printax	7	G	8,9747	12,9148	4,4482
Printax	8	G	9,9255	9,8029	7,6041
Printax	9	G	11,2923	13,1542	3,2125
Printax	10	G	9,7281	10,4203	5,4258
Printax	11	G	4,1360	6,6553	2,6776
Printax	12	G	4,7647	8,8916	5,4388
Printax	13	G	4,1064	4,9227	1,0377
Printax	14	G	6,8438	9,5343	4,4233
Printax	15	G	5,1474	7,6189	2,8571
Printax	16	G	4,8906	6,5550	1,7451
Printax	17	G	4,5623	7,7615	5,3514
Printax	18	G	5,8048	5,9501	2,5647
Printax	19	G	4,9927	8,6564	2,2274
Printax	20	G	4,8383	7,1607	3,3952
Printax	21	G	4,1765	1,4014	1,0690
Printax	22	G	5,0922	1,5523	1,9596
Printax	23	G	4,8352	0,5951	0,7218
Printax	24	G	6,6065	1,1896	1,8757
Printax	25	G	5,0277	1,4488	0,9347
Printax	26	G	4,5289	4,6563	2,1994
Printax	27	G	5,3136	1,4164	2,7810

Nanolab	25	PG	12,5085	1,8274	4,8427
Nanolab	26	PG	12,3625	1,8433	4,9423
Nanolab	27	PG	12,1132	2,1327	4,0956
Nanolab	28	PG	12,8971	2,2364	4,3265
Nanolab	29	PG	11,3739	1,8719	5,0955
Nanolab	30	PG	11,9357	1,5493	3,9523
Nanolab	1	G	14,8598	7,5836	1,3135
Nanolab	2	G	23,0973	8,6139	1,5191
Nanolab	3	G	21,9617	10,2740	3,0660
Nanolab	4	G	23,2487	10,8152	2,7046
Nanolab	5	G	24,1653	9,6785	0,8096
Nanolab	6	G	20,2449	8,8515	1,7857
Nanolab	7	G	25,6344	11,6029	3,7336
Nanolab	8	G	24,3402	9,6690	2,6619
Nanolab	9	G	20,2161	8,5609	0,9872
Nanolab	10	G	24,3693	4,4393	0,8689
Nanolab	11	G	16,7388	5,6869	2,6873
Nanolab	12	G	17,7384	6,0222	2,9473
Nanolab	13	G	16,1634	5,9647	2,7087
Nanolab	14	G	17,5676	12,6935	6,8585
Nanolab	15	G	17,1101	12,9889	6,5007
Nanolab	16	G	17,9165	5,5597	2,5387
Nanolab	17	G	18,4721	5,4156	2,5913
Nanolab	18	G	17,9995	4,7753	1,3489
Nanolab	19	G	17,2052	4,7162	2,5308
Nanolab	20	G	15,9150	4,7560	2,0265
Nanolab	21	G	12,2840	4,5264	8,1447
Nanolab	22	G	12,4928	3,6032	8,7014
Nanolab	23	G	11,9500	4,2055	8,0166
Nanolab	24	G	12,4056	5,3111	6,4549
Nanolab	25	G	11,8900	6,4769	6,7551
Nanolab	26	G	12,9497	2,9520	8,3420
Nanolab	27	G	11,4815	6,5272	10,8150

Printax	28	G	5,1354	0,8689	1,1151
Printax	29	G	4,4825	2,0727	4,6611
Printax	30	G	6,1825	0,4889	2,6078
Printax	1	POL	2,2365	1,9441	4,3137
Printax	2	POL	2,9109	1,0698	4,8170
Printax	3	POL	1,7550	0,9334	2,3270
Printax	4	POL	2,5406	2,1983	4,9665
Printax	5	POL	2,2671	0,7695	0,7579
Printax	6	POL	4,2965	0,5760	1,7906
Printax	7	POL	1,7447	1,0415	2,5792
Printax	8	POL	1,9784	0,6772	0,7377
Printax	9	POL	0,9861	2,6932	4,9088
Printax	10	POL	2,2150	2,7041	4,4625
Printax	11	POL	2,0501	1,3661	2,2506
Printax	12	POL	5,0284	1,8452	3,5141
Printax	13	POL	2,5322	0,8850	0,9792
Printax	14	POL	2,4780	0,6746	0,4295
Printax	15	POL	1,6354	1,3048	4,5398
Printax	16	POL	1,8598	1,4158	1,4278
Printax	17	POL	1,8855	2,1248	0,4717
Printax	18	POL	1,7391	1,3405	2,4021
Printax	19	POL	4,0830	2,3810	2,3577
Printax	20	POL	3,0394	2,5556	4,3698
Printax	21	POL	2,3108	2,5803	0,9151
Printax	22	POL	1,8793	1,4361	2,6934
Printax	23	POL	2,0717	0,7503	0,9842
Printax	24	POL	2,1487	1,8621	3,5806
Printax	25	POL	2,1780	0,9594	2,2446
Printax	26	POL	2,4639	1,4900	3,7351
Printax	27	POL	1,8928	0,7553	1,4156
Printax	28	POL	4,6592	0,7074	1,9315
Printax	29	POL	2,3604	1,4851	1,7210
Printax	30	POL	2,8018	1,7141	2,1955

Nanolab	28	G	12,2396	3,7000	7,8238
Nanolab	29	G	11,7221	5,5578	8,7701
Nanolab	30	G	11,7800	4,5216	9,8693
Nanolab	1	POL	16,9236	2,8925	1,0274
Nanolab	2	POL	16,3371	6,3152	0,7804
Nanolab	3	POL	18,3652	2,2481	0,6834
Nanolab	4	POL	18,0988	1,9770	1,4159
Nanolab	5	POL	16,3900	2,9269	1,7152
Nanolab	6	POL	17,7843	2,2961	0,1751
Nanolab	7	POL	17,2405	3,0328	0,9268
Nanolab	8	POL	17,7154	2,7599	1,1493
Nanolab	9	POL	16,6772	3,8395	2,6988
Nanolab	10	POL	18,6054	2,7926	1,0586
Nanolab	11	POL	14,5292	5,3648	2,3406
Nanolab	12	POL	15,8666	3,4033	2,0938
Nanolab	13	POL	15,8738	2,6262	1,7975
Nanolab	14	POL	16,3976	4,3413	3,0560
Nanolab	15	POL	16,7465	3,4763	2,2319
Nanolab	16	POL	16,8290	2,1259	1,2476
Nanolab	17	POL	17,5705	2,6922	0,7802
Nanolab	18	POL	14,3557	5,1734	0,9616
Nanolab	19	POL	16,2713	3,5032	1,5351
Nanolab	20	POL	15,6433	3,6868	1,9528
Nanolab	21	POL	12,5869	8,7293	11,2624
Nanolab	22	POL	15,8339	6,3010	7,9315
Nanolab	23	POL	11,5621	9,1726	11,4215
Nanolab	24	POL	10,8535	11,3239	11,2832
Nanolab	25	POL	12,0337	8,1285	10,7578
Nanolab	26	POL	12,1209	8,1042	10,7484
Nanolab	27	POL	10,3321	10,5634	11,9887
Nanolab	28	POL	9,4081	12,1267	12,2226
Nanolab	29	POL	12,0959	7,6009	10,8036
Nanolab	30	POL	12,4919	6,4963	10,9762

Resin	Sample	Subgroups	Δ1	Δ2	Δ3
Duralay	1	PG	12,8835	12,0036	1,4562
Duralay	2	PG	14,5862	7,7213	3,7006
Duralay	3	PG	14,347	12,8768	4,832
Duralay	4	PG	12,3967	5,9688	2,2623
Duralay	5	PG	8,8503	8,9317	1,5775
Duralay	6	PG	13,984	8,4221	4,9582
Duralay	7	PG	11,5666	10,6129	2,8293
Duralay	8	PG	12,9884	7,8056	2,6092
Duralay	9	PG	13,9857	13,2173	3,234
Duralay	10	PG	15,6185	9,5825	8,2686
Duralay	11	PG	8,7733	10,0904	3,1397
Duralay	12	PG	8,289	7,7177	1,8297
Duralay	13	PG	9,6395	8,9862	1,2047
Duralay	14	PG	9,4551	7,9871	1,0173
Duralay	15	PG	9,7037	7,643	1,2849
Duralay	16	PG	8,9885	6,1865	0,9873
Duralay	17	PG	8,6603	7,5238	1,0407
Duralay	18	PG	7,1663	6,9699	1,0889
Duralay	19	PG	9,7542	9,1628	3,8455
Duralay	20	PG	8,1773	6,3973	1,0365
Duralay	21	PG	1,3651	1,1263	3,0122
Duralay	22	PG	2,077	7,5497	8,2187
Duralay	23	PG	1,9992	0,9914	1,9824
Duralay	24	PG	1,4509	3,2919	5,1159

Duralay	25	PG	1,4893	6,4968	2,1551
Duralay	26	PG	2,3471	2,8729	3,9911
Duralay	27	PG	1,8464	5,5441	2,0756
Duralay	28	PG	2,0402	3,9977	3,3716
Duralay	29	PG	1,4083	10,1629	10,1286
Duralay	30	PG	3,1644	4,5826	1,1248
Duralay	1	G	11,257	7,6298	2,1726
Duralay	2	G	11,3273	6,5054	9,9117
Duralay	3	G	13,9978	9,3152	4,2565
Duralay	4	G	21,3562	17,2414	2,1724
Duralay	5	G	12,7595	10,9398	1,2646
Duralay	6	G	13,2981	7,6923	1,1864
Duralay	7	G	10,0076	9,7615	1,0468
Duralay	8	G	12,8802	5,8923	1,184
Duralay	9	G	11,4939	9,6377	1,0847
Duralay	10	G	15,3051	7,5315	1,9213
Duralay	11	G	7,7953	5,7792	1,6291
Duralay	12	G	8,2708	5,6043	2,2653
Duralay	13	G	6,7283	4,4185	1,4318
Duralay	14	G	8,8509	8,0824	2,5314
Duralay	15	G	6,9991	6,2321	1,7597
Duralay	16	G	7,3699	6,6155	1,3372
Duralay	17	G	6,9912	5,2446	2,084
Duralay	18	G	8,5303	6,6915	1,0906
Duralay	19	G	9,1262	7,2076	1,2775
Duralay	20	G	7,7689	5,9122	2,6532
Duralay	21	G	0,9605	1,493	0,95
Duralay	22	G	1,1661	0,7055	2,0049
Duralay	23	G	1,0599	3,1571	0,6752
Duralay	24	G	1,1085	0,9368	2,4691
Duralay	25	G	1,752	3,0935	1,8825
Duralay	26	G	0,9587	2,2496	3,0919
Duralay	27	G	1,5513	3,0117	2,4407

Duralay	28	G	2,6932	1,0088	0,6441
Duralay	29	G	1,4288	2,1744	0,5968
Duralay	30	G	3,5911	5,182	1,4544
Duralay	1	POL	2,222	0,4392	6,5897
Duralay	2	POL	1,807	1,3038	0,8328
Duralay	3	POL	12,3671	0,6023	1,6838
Duralay	4	POL	1,5155	2,504	0,7213
Duralay	5	POL	1,1591	4,4846	1,9527
Duralay	6	POL	3,0347	5,7233	1,0765
Duralay	7	POL	1,9562	2,182	1,5186
Duralay	8	POL	1,1772	2,0497	1,9852
Duralay	9	POL	1,8844	2,2333	2,7551
Duralay	10	POL	2,177	1,0048	0,5496
Duralay	11	POL	1,1389	2,9198	7,515
Duralay	12	POL	1,2916	1,1176	0,4602
Duralay	13	POL	2,8896	5,5445	0,7459
Duralay	14	POL	1,0805	2,4722	2,1509
Duralay	15	POL	1,5289	1,3166	2,1937
Duralay	16	POL	1,4796	1,1973	5,5435
Duralay	17	POL	0,8763	3,7659	1,5003
Duralay	18	POL	2,137	8,3026	0,9678
Duralay	19	POL	1,0891	4,3521	9,3131
Duralay	20	POL	7,6923	9,0504	8,7446
Duralay	21	POL	3,0079	0,6258	1,5009
Duralay	22	POL	0,5737	2,2641	2,5662
Duralay	23	POL	1,0517	1,1892	1,2242
Duralay	24	POL	0,7943	4,3942	3,2013
Duralay	25	POL	2,2827	4,4695	3,6729
Duralay	26	POL	1,5868	4,8252	1,2359
Duralay	27	POL	0,7863	1,0581	1,6727
Duralay	28	POL	1,5823	3,8542	1,3476
Duralay	29	POL	1,0442	2,2672	2,945
Duralay	30	POL	0,871	0,5842	0,4521
