# Supplementary material for: 3D-Printed Versus Conventional Dental Provisional Resins: A Comparative Study
Source: Medicina (Kaunas). 2026 Feb 14;62(2):382. doi: 10.3390/medicina62020382 (PMC12942832; doi:10.3390/medicina62020382)
Supplement: Supplementary file 1 [file medicina-62-00382-s001.zip › S2_Knoop Microhardness.pdf]

Knoop Microhardness

1-10: Immersed in cola soft drink

11-20: Immersed in energy drink

21-30: Immersed in distilled water

Δ1: T0-T1 (Initial - After 1st Immersion)

Δ2: T1-T2 (After 1st Immersion - After Brushing)

Δ2: T1-T3 (After Brushing - After 2nd Immersion)

Resin	Sample	Subgroups	Δ1	Δ2	Δ3
Printax	1	PG	-6,314	-1,204	-0,498
Printax	2	PG	-6,47	-0,658	-0,688
Printax	3	PG	-3,794	-2,904	-0,128
Printax	4	PG	1,16	-6,504	0,506
Printax	5	PG	-6,036	0,076	-1,696
Printax	6	PG	-6,04	-1,742	0,72
Printax	7	PG	-3,442	-0,64	0,698
Printax	8	PG	-4,472	-1,384	0,084
Printax	9	PG	-3,676	-3,418	1,246
Printax	10	PG	0,352	-5,972	1,222
Printax	11	PG	1,000	0,36	-1,78
Printax	12	PG	-1,288	-3,018	5,086
Printax	13	PG	1,626	-6,354	3,528
Printax	14	PG	-1,96	-3,024	1,702
Printax	15	PG	2,74	-4,788	2,266
Printax	16	PG	-0,146	-1,538	3,318
Printax	17	PG	-1,668	-2,646	3,988
Printax	18	PG	-4,108	0,712	0,894
Printax	19	PG	-4,29	-1,094	4,324
Printax	20	PG	-0,6	-2,382	0,076
Printax	21	PG	-1,212	-2,582	2,45
Printax	22	PG	-1,984	1,444	-2,108
Printax	23	PG	-1,212	-2,262	0,228
Printax	24	PG	-0,526	-2,594	0,082

Resin	Sample	Subgroups	Δ1	Δ2	Δ3
Nanolab	1	PG	0,105	5,26	-4,9725
Nanolab	2	PG	0,125	1,495	0,77
Nanolab	3	PG	1,3175	-2,8975	1,0825
Nanolab	4	PG	0,5975	-1,3575	1,4275
Nanolab	5	PG	-0,715	0,13	-0,085
Nanolab	6	PG	0,5275	1,4075	0,17
Nanolab	7	PG	-1,825	0,32	2,0325
Nanolab	8	PG	-2,0575	0,6	0,7425
Nanolab	9	PG	1,29	0,4325	0,8475
Nanolab	10	PG	3,125	-2,375	0,225
Nanolab	11	PG	-0,4275	0,51	0,0125
Nanolab	12	PG	4,235	-3,4125	-0,13
Nanolab	13	PG	2,105	-1,4725	-0,3025
Nanolab	14	PG	1,74	-2,095	1,1
Nanolab	15	PG	4,5575	-3,17	-0,9475
Nanolab	16	PG	-0,165	0,615	0,665
Nanolab	17	PG	2,9975	-2,6275	-0,655
Nanolab	18	PG	6,03	-3,51	-0,635
Nanolab	19	PG	2,9575	-4,3025	1,6
Nanolab	20	PG	2,045	-3,845	1,1875
Nanolab	21	PG	-1,0825	-1,9825	0,98
Nanolab	22	PG	2,305	-3,7325	1,8875
Nanolab	23	PG	2,89	-4,395	1,43
Nanolab	24	PG	3,5225	-2,43	0,4475

Printax	25	PG	-0,244	-1,558	0,09
Printax	26	PG	-1,704	-1,342	-0,356
Printax	27	PG	-0,012	-2,686	1,446
Printax	28	PG	-0,618	-1,996	0,44
Printax	29	PG	-2,182	-1,262	-0,046
Printax	30	PG	1,272	-4,136	-1,452
Printax	1	G	5,126	-7,384	1,136
Printax	2	G	-0,896	-2,008	-1,396
Printax	3	G	2,074	-2,508	-0,84
Printax	4	G	-1,568	-0,902	-0,326
Printax	5	G	-3,43	1,546	2,644
Printax	6	G	1,736	-3,616	1,914
Printax	7	G	2,164	0,584	-1,806
Printax	8	G	0,202	-0,048	-0,134
Printax	9	G	1,508	5,59	-1,58
Printax	10	G	5,1	-4,49	1,686
Printax	11	G	-2,376	0,106	0,352
Printax	12	G	-1,016	-2,472	1,828
Printax	13	G	5,2	-3,864	0,506
Printax	14	G	0,336	0,376	-1,758
Printax	15	G	0,358	-3,19	0,782
Printax	16	G	0,028	-3,578	4,59
Printax	17	G	-4,902	-2,394	3,226
Printax	18	G	-5,446	-1,378	4,494
Printax	19	G	0,21	-4,56	5,496
Printax	20	G	-5,174	-4,446	3,776
Printax	21	G	1,2	-0,232	-3,828
Printax	22	G	1,156	-4,084	0,954
Printax	23	G	3,204	-1,388	-3,166
Printax	24	G	3,35	-5,328	3,946
Printax	25	G	0,028	-2,76	2,896
Printax	26	G	4,528	-6,94	1,672
Printax	27	G	-1,88	0,904	-1,384

Nanolab	25	PG	4,495	-6,145	3,1525
Nanolab	26	PG	2,1825	-3,935	1,9525
Nanolab	27	PG	4,1625	-4,1475	1,6525
Nanolab	28	PG	1,0475	-3,39	1,86
Nanolab	29	PG	0,0925	-1,0275	1,51
Nanolab	30	PG	2,995	-3,375	1,3775
Nanolab	1	G	-0,1675	0,06	0,445
Nanolab	2	G	0,0675	-2,3425	1,845
Nanolab	3	G	-0,5675	-2,825	2,14
Nanolab	4	G	0,745	-0,1975	-0,075
Nanolab	5	G	-0,71	-3,0525	2,135
Nanolab	6	G	-3,155	-1,98	1,36
Nanolab	7	G	-3,09	-0,7375	0,9525
Nanolab	8	G	-0,6	-1,9225	1,275
Nanolab	9	G	-0,2025	-3,2075	0,5225
Nanolab	10	G	2,28	-1,225	0,635
Nanolab	11	G	3,9325	-3,2825	1,2875
Nanolab	12	G	1,3575	-4,5425	1,205
Nanolab	13	G	1,85	-4,6575	1,83
Nanolab	14	G	-1,0925	-2,7625	0,2375
Nanolab	15	G	0,4	-3,46	0,7525
Nanolab	16	G	-2,2325	-1,4625	1,06
Nanolab	17	G	-0,83	-2,7025	0,8625
Nanolab	18	G	0,845	-2,9575	0,3675
Nanolab	19	G	-0,96	-3,2775	0,9275
Nanolab	20	G	-3,3	-2,675	-1,195
Nanolab	21	G	2,765	-5,7775	1,965
Nanolab	22	G	3,0175	-4,735	1,3875
Nanolab	23	G	-4,7375	-1,4375	0,965
Nanolab	24	G	-1,9125	-3,535	1,615
Nanolab	25	G	1,545	-3,0975	1,645
Nanolab	26	G	0,4525	-3,97	1,08
Nanolab	27	G	0,995	-3,5675	-1,53

Printax	28	G	-0,92	-3,092	-1,174
Printax	29	G	1,4	-3,3	-1,466
Printax	30	G	6,134	-4,14	-3,808
Printax	1	POL	-0,38	1,64	-9,39
Printax	2	POL	-5,72	-5,212	-3,406
Printax	3	POL	0,22	0,76	-5,59
Printax	4	POL	-3,314	0,894	-3,588
Printax	5	POL	-2,02	3,24	-5,178
Printax	6	POL	-6,206	1,214	-3,452
Printax	7	POL	0,124	3,056	-7,514
Printax	8	POL	-4,64	0,14	-5,264
Printax	9	POL	-3,4	1,36	-5,238
Printax	10	POL	-0,64	0,78	-4,41
Printax	11	POL	-0,74	2,14	-2,66
Printax	12	POL	6,13	-0,43	-6,618
Printax	13	POL	2,446	2,86	-4,18
Printax	14	POL	0,74	3,02	-9,48
Printax	15	POL	-2,86	1,28	-3,6
Printax	16	POL	2,22	-0,48	-2,22
Printax	17	POL	2,54	1,48	-6,122
Printax	18	POL	2,08	2,6	-7,42
Printax	19	POL	0,38	3,52	-8,1
Printax	20	POL	1,32	3,72	-7,72
Printax	21	POL	1,08	-1,604	-4,692
Printax	22	POL	-1,2	-1,4	-3,32
Printax	23	POL	4,62	-3,2	-3,36
Printax	24	POL	0,3	-0,98	-3,896
Printax	25	POL	-0,04	0,22	-4,606
Printax	26	POL	-0,48	-0,64	-4,078
Printax	27	POL	2,66	-2,34	-2,7
Printax	28	POL	3,7	-5,58	-0,4
Printax	29	POL	4,36	-5,74	-6,9
Printax	30	POL	4,6	-1,58	-9,492

Nanolab	28	G	-2,68	-1,4	0,26
Nanolab	29	G	-0,7875	-3,57	0,025
Nanolab	30	G	-4,79	-1,6725	-0,34
Nanolab	1	POL	-3,45	-4,6975	-0,245
Nanolab	2	POL	5,28	-1,99	-0,395
Nanolab	3	POL	-0,025	-6,5175	-0,255
Nanolab	4	POL	-3,275	-2,645	-0,845
Nanolab	5	POL	-3,9	-3,6075	-1,9975
Nanolab	6	POL	-2,675	-2,635	-2,515
Nanolab	7	POL	-2,4	-4,755	-1,54
Nanolab	8	POL	2,24	-3,575	-1,6725
Nanolab	9	POL	-4,75	-1,34	-2,6275
Nanolab	10	POL	-3,25	-2,1875	-3,275
Nanolab	11	POL	-2,075	-2,8325	-2,5025
Nanolab	12	POL	-3,3	-1,145	-2,3775
Nanolab	13	POL	-7,3	-2,0425	-1,445
Nanolab	14	POL	-1,525	-3,6	-1,9775
Nanolab	15	POL	2,4875	-2,9	-2,8025
Nanolab	16	POL	0,05	-0,265	-3,415
Nanolab	17	POL	-2,075	-3,0075	-1,9225
Nanolab	18	POL	0,3125	-2,355	-3,135
Nanolab	19	POL	-2,125	-0,8075	-3,95
Nanolab	20	POL	1,0875	-2,3775	-3,28
Nanolab	21	POL	0,175	-5,435	-0,7825
Nanolab	22	POL	-5,15	-4,4725	-0,2625
Nanolab	23	POL	2,4925	-3,815	-0,31
Nanolab	24	POL	-3,6475	-2,355	-1,4725
Nanolab	25	POL	-5,67	-2,38	-0,725
Nanolab	26	POL	-3,075	-3,8975	-0,8525
Nanolab	27	POL	-4,025	-3,2075	-1,1025
Nanolab	28	POL	2,005	-1,215	-0,2575
Nanolab	29	POL	-6,35	-3,6325	-0,9325
Nanolab	30	POL	-2,85	-2,3625	-1,9625

Resin	Sample	Subgroups	Δ1	Δ2	Δ3
Duralay	1	PG	-1,5395	-0,884	0,18
Duralay	2	PG	-2,061	-0,666	0,664
Duralay	3	PG	-2,0315	-1,408	1,2
Duralay	4	PG	-1,519	-1,992	0,584
Duralay	5	PG	-2,2555	-0,104	-0,334
Duralay	6	PG	-1,352	-0,79	0,036
Duralay	7	PG	-1,6155	-1,16	0,93
Duralay	8	PG	-1,8605	-0,72	0,71
Duralay	9	PG	-1,752	-1,71	0,61
Duralay	10	PG	-2,4885	0,32	0,17
Duralay	11	PG	-0,0265	-2,496	0,102
Duralay	12	PG	-0,459	-2,364	0,036
Duralay	13	PG	-0,072	-1,934	0,438
Duralay	14	PG	-0,3105	-0,956	-0,238
Duralay	15	PG	-1,831	-0,996	0,73
Duralay	16	PG	-2,246	-1,828	1,264
Duralay	17	PG	-2,558	-0,392	-0,268
Duralay	18	PG	-1,55	-0,07	-0,386
Duralay	19	PG	-1,974	-0,658	0,366
Duralay	20	PG	-0,723	-1,956	1,284
Duralay	21	PG	1,49	-2,324	-0,052
Duralay	22	PG	-0,8605	-0,692	0,872
Duralay	23	PG	0,9555	-2,852	0,116
Duralay	24	PG	0,923	-2,186	-0,132

Duralay	25	PG	-0,5045	-2,16	1,258
Duralay	26	PG	-0,3165	-2,05	0,796
Duralay	27	PG	-0,455	-3,484	1,37
Duralay	28	PG	1,0195	-2,942	0,526
Duralay	29	PG	0,38	-2,866	0,794
Duralay	30	PG	1,135	-4,066	1,048
Duralay	1	G	-1,32	-0,798	0,664
Duralay	2	G	-2,004	-0,024	0,694
Duralay	3	G	-1,55	-0,684	0,618
Duralay	4	G	-1,536	-0,554	0,716
Duralay	5	G	-0,328	-1,488	0,26
Duralay	6	G	-1,556	-1,33	0,902
Duralay	7	G	-1,368	-0,878	0,552
Duralay	8	G	-1,118	-0,752	0,536
Duralay	9	G	-1,364	-1,434	1,132
Duralay	10	G	-1,878	-0,254	-0,126
Duralay	11	G	-0,298	-1,932	1,674
Duralay	12	G	-0,912	-2,388	1,322
Duralay	13	G	-1,48	-1,182	1,258
Duralay	14	G	-0,788	-2,552	1,038
Duralay	15	G	-2	-0,934	-0,22
Duralay	16	G	-0,914	-2,048	-0,012
Duralay	17	G	-1,728	-1,188	-0,198
Duralay	18	G	-1,764	-2,308	1,452
Duralay	19	G	-1,984	-2,618	1,562
Duralay	20	G	-0,256	-2,762	1,824
Duralay	21	G	0,08	-2,342	0,458
Duralay	22	G	0,038	-1,956	-0,644
Duralay	23	G	0,3	-2,538	0,12
Duralay	24	G	0,344	-2,246	0,638
Duralay	25	G	0,232	-1,63	-0,216
Duralay	26	G	-2,444	-0,404	0,258
Duralay	27	G	-0,724	-2,786	0,378

Duralay	28	G	2,16	-5,148	1,484
Duralay	29	G	0,1	-2,6	0,506
Duralay	30	G	0,464	-3,978	0,94
Duralay	1	POL	0,322	-2,296	0,086
Duralay	2	POL	0,61	-3,642	0,296
Duralay	3	POL	0,11	-4,12	0,954
Duralay	4	POL	-1,475	-1,996	0,388
Duralay	5	POL	-0,17	-3,442	0,726
Duralay	6	POL	0,045	-3,174	-0,112
Duralay	7	POL	-0,428	-3,542	0,838
Duralay	8	POL	-0,107	-3,608	0,572
Duralay	9	POL	0,472	-2,694	-0,694
Duralay	10	POL	1,605	-3,668	-1,512
Duralay	11	POL	0,234	-1,67	-0,834
Duralay	12	POL	0,116	-2,062	-0,106
Duralay	13	POL	1,062	-3,3	0,322
Duralay	14	POL	1,42	-3,658	-0,182
Duralay	15	POL	1,73	-4,9	0,474
Duralay	16	POL	0,855	-3,656	0,872
Duralay	17	POL	0,9725	-3,424	0,17
Duralay	18	POL	2,1975	-3,656	-0,28
Duralay	19	POL	0,68	-3,338	-0,314
Duralay	20	POL	0,905	-3,654	-0,802
Duralay	21	POL	0,209	-3,938	1,484
Duralay	22	POL	-0,81	-3,726	1,332
Duralay	23	POL	0,975	-3,27	-0,574
Duralay	24	POL	-0,241	-2,45	-0,648
Duralay	25	POL	0,125	-3,982	-0,772
Duralay	26	POL	0,665	-3,73	-0,114
Duralay	27	POL	0,64	-4,098	0,08
Duralay	28	POL	0,505	-3,884	0,424
Duralay	29	POL	1,637	-3,02	-0,852
Duralay	30	POL	0,7275	-2,812	0,392
