# Supplementary material for: 3D-Printed Versus Conventional Dental Provisional Resins: A Comparative Study
Source: Medicina (Kaunas). 2026 Feb 14;62(2):382. doi: 10.3390/medicina62020382 (PMC12942832; doi:10.3390/medicina62020382)
Supplement: Supplementary file 1 [file medicina-62-00382-s001.zip › S3_Roughness.pdf]

Roughness

1-10: Immersed in cola soft drink
11-20: Immersed in energy drink
21-30: Immersed in distilled water

Δ1: T0-T1 (Initial - After 1st Immersion)
Δ2: T1-T2 (After 1st Immersion - After Brushing)
Δ2: T1-T3 (After Brushing - After 2nd Immersion)

Resin	Sample	Subgroups	Δ1	Δ2	Δ3
Printax	1	PG	-0,671	-0,328	0,334
Printax	2	PG	0,652	-0,495	0,314
Printax	3	PG	0,816	-0,982	1,799
Printax	4	PG	2,495	-2,023	0,939
Printax	5	PG	0,227	-0,313	0,559
Printax	6	PG	0,296	0,227	0,567
Printax	7	PG	0,561	-0,353	0,67
Printax	8	PG	0,484	-0,562	0,97
Printax	9	PG	0,984	-0,831	0,854
Printax	10	PG	-0,125	-0,493	0,763
Printax	11	PG	3,615	-2,76	0,871
Printax	12	PG	1,485	-0,828	0,819
Printax	13	PG	0,676	-0,494	1,67
Printax	14	PG	-0,387	-0,377	0,585
Printax	15	PG	0,083	-0,019	0,658
Printax	16	PG	0,031	0,266	0,89
Printax	17	PG	-0,036	0,6	0,242
Printax	18	PG	0,063	0,242	0,353
Printax	19	PG	0,219	0,018	2,055
Printax	20	PG	4,979	-5,018	0,56
Printax	21	PG	-0,081	0,554	0,615
Printax	22	PG	-0,075	0,271	1,706
Printax	23	PG	0,013	0,483	0,461
Printax	24	PG	0,159	0,244	0,477
Printax	25	PG	-1,019	0,441	1,29

Resin	Sample	Subgroups	Δ1	Δ2	Δ3
Nanolab	1	PG	-0,642	-2,355	0,339
Nanolab	2	PG	-0,386	-0,354	0,073
Nanolab	3	PG	-0,786	-0,211	-0,027
Nanolab	4	PG	-0,584	0,369	0,088
Nanolab	5	PG	0,537	-1,712	0,108
Nanolab	6	PG	0,079	-0,518	-0,1
Nanolab	7	PG	-0,095	-0,4	0,063
Nanolab	8	PG	-0,047	-0,216	0,049
Nanolab	9	PG	0,014	-0,521	0,124
Nanolab	10	PG	-0,464	-0,12	0,167
Nanolab	11	PG	-1,422	-1,759	-0,222
Nanolab	12	PG	0,118	-1,485	-0,169
Nanolab	13	PG	0,016	0,208	-0,118
Nanolab	14	PG	-0,079	1,01	0,016
Nanolab	15	PG	-0,873	-1,332	0,112
Nanolab	16	PG	0,141	-1,841	0,02
Nanolab	17	PG	-0,847	-1,319	-0,081
Nanolab	18	PG	-0,305	0,085	-0,148
Nanolab	19	PG	-0,242	-0,183	-0,105
Nanolab	20	PG	-0,105	-0,245	0,469
Nanolab	21	PG	-0,253	0,073	0,058
Nanolab	22	PG	-0,094	0,353	0,139
Nanolab	23	PG	-0,676	0,246	-0,049
Nanolab	24	PG	0,589	-1,19	-0,119
Nanolab	25	PG	0,41	-0,711	0,227

Printax	26	PG	-0,011	0,741	0,281
Printax	27	PG	-0,089	0,059	0,99
Printax	28	PG	-0,07	0,731	1,028
Printax	29	PG	0,282	-0,152	0,838
Printax	30	PG	-0,08	0,461	1,341
Printax	1	G	0,053	0,648	0,826
Printax	2	G	-0,273	0,442	1,128
Printax	3	G	-0,152	1,02	0,64
Printax	4	G	0,147	0,83	1,005
Printax	5	G	0,079	0,972	0,434
Printax	6	G	0,333	0,153	0,825
Printax	7	G	8,101	-6,463	0,252
Printax	8	G	0,113	0,123	1,623
Printax	9	G	0,835	0,582	0,639
Printax	10	G	0,092	0,535	0,572
Printax	11	G	0,249	0,444	0,821
Printax	12	G	0,632	0,56	0,223
Printax	13	G	0,484	0,938	-0,181
Printax	14	G	0,415	0,931	0,209
Printax	15	G	-0,029	0,923	0,279
Printax	16	G	0,725	0,08	0,832
Printax	17	G	0,257	0,39	0,64
Printax	18	G	0,114	0,549	1,617
Printax	19	G	0,001	0,939	1,634
Printax	20	G	0,609	0,537	0,686
Printax	21	G	0,001	0,915	0,504
Printax	22	G	0,006	1,296	0,282
Printax	23	G	0,111	1,057	0,466
Printax	24	G	0,017	0,98	0,959
Printax	25	G	0,156	1,1	0,325
Printax	26	G	0,159	0,593	0,133
Printax	27	G	0,966	0,399	0,328
Printax	28	G	0,007	0,845	1,414

Nanolab	26	PG	-0,095	0,246	0,002
Nanolab	27	PG	-0,342	0,21	0,43
Nanolab	28	PG	-0,255	0,536	-0,436
Nanolab	29	PG	-0,372	0,063	0,003
Nanolab	30	PG	-0,342	0,121	-0,315
Nanolab	1	G	0,02	0,231	0,236
Nanolab	2	G	-0,177	-0,03	0,663
Nanolab	3	G	-0,092	-0,149	0,512
Nanolab	4	G	-0,23	0,505	-0,125
Nanolab	5	G	-0,063	0,312	0,376
Nanolab	6	G	-0,172	1,099	-0,073
Nanolab	7	G	-0,092	0,225	0,272
Nanolab	8	G	-0,371	0,408	-0,163
Nanolab	9	G	-0,071	0,555	-0,157
Nanolab	10	G	-0,059	0,544	-0,067
Nanolab	11	G	0,093	0,085	-0,03
Nanolab	12	G	-0,083	0,429	0,075
Nanolab	13	G	0,019	0,037	0,262
Nanolab	14	G	0,102	0,605	-0,064
Nanolab	15	G	-0,02	0,414	-0,175
Nanolab	16	G	0,371	-0,005	0,059
Nanolab	17	G	-0,088	-0,617	0,592
Nanolab	18	G	-0,044	1,38	-0,517
Nanolab	19	G	-0,108	0,269	-0,128
Nanolab	20	G	0,01	0,236	-0,13
Nanolab	21	G	0,322	0,453	-0,04
Nanolab	22	G	0,013	0,617	-0,064
Nanolab	23	G	0,043	0,477	-0,45
Nanolab	24	G	-0,196	0,653	-0,071
Nanolab	25	G	-0,109	0,763	-0,214
Nanolab	26	G	-0,315	0,667	0,036
Nanolab	27	G	-0,326	0,524	-0,009
Nanolab	28	G	-0,019	0,705	-0,013

Printax	29	G	0,164	1,379	0,22
Printax	30	G	0,175	0,759	0,496
Printax	1	POL	16,824	-18,743	0,283
Printax	2	POL	12,255	-16,956	0,792
Printax	3	POL	13,228	-14,431	0,37
Printax	4	POL	16,296	-17,378	0,619
Printax	5	POL	16,498	-18,737	0,498
Printax	6	POL	25,865	-30,799	1,573
Printax	7	POL	17,973	-19,586	0,795
Printax	8	POL	6,807	-8,839	1,81
Printax	9	POL	11,488	-15,585	1,679
Printax	10	POL	12,694	-14,097	0,533
Printax	11	POL	1,461	-4,961	1,407
Printax	12	POL	15,363	-18,883	0,372
Printax	13	POL	-3,489	-1,153	0,363
Printax	14	POL	-2,005	-0,629	1,696
Printax	15	POL	-2,881	-0,607	1,582
Printax	16	POL	-5,647	-0,677	0,751
Printax	17	POL	-7,155	-0,268	-0,231
Printax	18	POL	-4,719	-1,236	-0,168
Printax	19	POL	-8,678	-1,367	0,422
Printax	20	POL	-5,309	-1,384	-0,461
Printax	21	POL	-13,154	-0,672	0,635
Printax	22	POL	-9,113	-1,831	0,357
Printax	23	POL	-3,024	-5,232	0,46
Printax	24	POL	-3,212	-4,032	0,576
Printax	25	POL	-6,573	-4,291	0,08
Printax	26	POL	-2,233	-5,301	-0,045
Printax	27	POL	1,977	-4,662	-0,122
Printax	28	POL	0,504	-2,505	0,027
Printax	29	POL	-5,857	-3,769	0,306
Printax	30	POL	4,353	-8,292	-0,093

Nanolab	29	G	-0,189	0,767	-0,101
Nanolab	30	G	0,35	-0,738	0,086
Nanolab	1	POL	-0,061	-0,787	0,58
Nanolab	2	POL	-0,952	0,372	-0,169
Nanolab	3	POL	-0,596	0,553	-0,018
Nanolab	4	POL	-0,313	0,546	-0,155
Nanolab	5	POL	-0,152	0,482	-0,091
Nanolab	6	POL	-0,263	0,494	0,183
Nanolab	7	POL	-0,324	0,677	-0,294
Nanolab	8	POL	-0,279	0,82	-0,184
Nanolab	9	POL	-0,289	0,522	0,101
Nanolab	10	POL	-0,309	0,871	-0,329
Nanolab	11	POL	-0,041	0,172	-0,173
Nanolab	12	POL	0,13	0,194	-0,087
Nanolab	13	POL	0,116	0,191	-0,275
Nanolab	14	POL	-0,016	0,502	-0,138
Nanolab	15	POL	-0,069	0,363	-0,162
Nanolab	16	POL	-0,109	0,37	-0,182
Nanolab	17	POL	0,174	0,413	-0,078
Nanolab	18	POL	-0,733	1,026	0,474
Nanolab	19	POL	-0,234	0,514	0,139
Nanolab	20	POL	-0,18	0,343	-0,236
Nanolab	21	POL	-0,187	0,253	-0,147
Nanolab	22	POL	-0,178	0,517	-0,414
Nanolab	23	POL	-0,21	0,358	-0,278
Nanolab	24	POL	-0,08	0,623	-0,472
Nanolab	25	POL	-0,182	0,593	-0,205
Nanolab	26	POL	-0,049	0,451	-0,339
Nanolab	27	POL	-0,254	0,805	-0,296
Nanolab	28	POL	0,083	0,604	-0,382
Nanolab	29	POL	-0,454	0,479	-0,062
Nanolab	30	POL	-0,096	0,292	0,005

Resin	Sample	Subgroups	Δ1	Δ2	Δ3
Duralay	1	PG	-0,904	-1,352	0,336
Duralay	2	PG	-0,868	0,588	0,253
Duralay	3	PG	-0,676	0,181	-0,107
Duralay	4	PG	-1,37	-0,162	0,219
Duralay	5	PG	-1,19	-0,358	-0,171
Duralay	6	PG	-0,414	0,382	0,229
Duralay	7	PG	0,058	0,136	-0,19
Duralay	8	PG	-0,03	0,299	-0,116
Duralay	9	PG	-0,712	-0,537	0,016
Duralay	10	PG	-0,318	1,486	-0,075
Duralay	11	PG	-0,035	-0,146	0,238
Duralay	12	PG	0,134	-0,009	-0,354
Duralay	13	PG	-0,019	0,083	-0,515
Duralay	14	PG	-0,958	-0,633	0,432
Duralay	15	PG	-0,312	-0,386	0,141
Duralay	16	PG	1,064	-1,503	0,636
Duralay	17	PG	-0,734	-0,696	0,1
Duralay	18	PG	-3,863	-0,233	0,587
Duralay	19	PG	-0,314	-0,415	0,235
Duralay	20	PG	-0,023	-0,53	0,324
Duralay	21	PG	0,298	0,462	-0,047
Duralay	22	PG	-1,442	0,803	0,18
Duralay	23	PG	1,383	-1,642	2,167
Duralay	24	PG	-2,569	-0,632	0,49
Duralay	25	PG	-0,171	-0,317	0,295

Duralay	26	PG	-3,132	-0,764	-0,149
Duralay	27	PG	-1,617	-0,365	0,205
Duralay	28	PG	-0,508	-0,474	0,2
Duralay	29	PG	-0,138	-0,141	0,375
Duralay	30	PG	-0,351	-0,574	-0,273
Duralay	1	G	-0,298	-0,884	0,634
Duralay	2	G	-1,732	-0,366	0,182
Duralay	3	G	-2,023	0,601	0,342
Duralay	4	G	0,29	-0,136	0,565
Duralay	5	G	-0,82	0,016	0,071
Duralay	6	G	-0,886	-1,124	0,301
Duralay	7	G	0,35	-0,367	0,226
Duralay	8	G	-0,7	-0,49	-0,021
Duralay	9	G	-0,869	-0,432	0,247
Duralay	10	G	-1,127	-0,281	-0,034
Duralay	11	G	-0,211	-0,682	-0,154
Duralay	12	G	0,069	-0,308	-0,573
Duralay	13	G	0,307	-0,817	0,174
Duralay	14	G	-0,105	0,061	-0,014
Duralay	15	G	-0,139	-0,636	-0,151
Duralay	16	G	0,175	-0,11	0,305
Duralay	17	G	1,546	-1,689	1,142
Duralay	18	G	0,128	-0,14	0,17
Duralay	19	G	0,179	-0,385	-0,045
Duralay	20	G	0,034	-0,576	-0,095
Duralay	21	G	0,255	-0,403	0,282
Duralay	22	G	-0,912	-0,231	0,145
Duralay	23	G	-0,395	-0,168	0,217
Duralay	24	G	-0,668	0,195	0,057
Duralay	25	G	-0,829	-0,25	-0,532
Duralay	26	G	-0,544	0,636	-0,025
Duralay	27	G	-0,503	0,211	-0,165
Duralay	28	G	-1,521	0,496	-0,038

Duralay	29	G	-0,497	-0,346	-0,203
Duralay	30	G	-1,04	-0,191	0,262
Duralay	1	POL	0,212	-0,381	0,438
Duralay	2	POL	0,285	-0,908	-0,025
Duralay	3	POL	-0,141	0,204	0,397
Duralay	4	POL	-0,256	0,362	0,052
Duralay	5	POL	0,123	0,238	-0,251
Duralay	6	POL	0,079	-0,284	0,037
Duralay	7	POL	-0,173	0,356	0,031
Duralay	8	POL	0,282	1,343	-0,488
Duralay	9	POL	0,571	-0,248	1,171
Duralay	10	POL	0,129	-0,799	-0,143
Duralay	11	POL	-0,175	-0,086	0,113
Duralay	12	POL	-0,094	-0,058	-0,083
Duralay	13	POL	0,01	-0,341	-0,173
Duralay	14	POL	1,206	-0,836	-0,097
Duralay	15	POL	0,29	0,692	-0,201
Duralay	16	POL	-0,373	2,348	0,021
Duralay	17	POL	-0,096	-0,741	-0,134
Duralay	18	POL	-0,633	3,413	1,117
Duralay	19	POL	-0,084	-0,289	0,197
Duralay	20	POL	0,611	3,008	0,819
Duralay	21	POL	-0,379	-0,026	0,262
Duralay	22	POL	-0,052	0,379	0,087
Duralay	23	POL	-0,124	0,127	-0,203
Duralay	24	POL	0,026	0,105	0,79
Duralay	25	POL	0,177	-0,674	0,722
Duralay	26	POL	0,123	0,261	-0,309
Duralay	27	POL	-0,018	-0,141	0,144
Duralay	28	POL	0,527	-0,852	0,477
Duralay	29	POL	-0,398	-0,809	0,028
Duralay	30	POL	0,661	-0,064	-0,18
